# Supplementary material for: The Systems Biology Research Tool: evolvable open-source software
Source: BMC Syst Biol. 2008 Jun 29;2:55. doi: 10.1186/1752-0509-2-55 (PMC2446383; doi:10.1186/1752-0509-2-55)
Supplement: Additional file 1 — SBRT Archive. An archive of the current version of the Systems Biology Research Tool. [file 1752-0509-2-55-S1.zip › sbrt-1.4.0/doc/developers_guide/api/sbrt/shell/gui/TextPaneOutputStream.html]

TextPaneOutputStream


|  |  |  |
| --- | --- | --- |
| **Overview** \| **Package** \| **Class** \| **Use** \| **Tree** \| **Deprecated** \| **Index** \| **Help** | |  |
| **PREV CLASS**   NEXT CLASS | **FRAMES**    **NO FRAMES**     **All Classes** |  |
| SUMMARY: NESTED \| FIELD \| CONSTR \| METHOD | DETAIL: FIELD \| CONSTR \| METHOD |  |


---


## sbrt.shell.gui Class TextPaneOutputStream

```
java.lang.Object
  java.io.OutputStream
      sbrt.shell.gui.TextPaneOutputStream
```

**All Implemented Interfaces:**: java.io.Closeable, java.io.Flushable

---

``` public class TextPaneOutputStream extends java.io.OutputStream ```

An output stream that writes its output to a javax.swing.JTextArea
control.

**Author:**
:   Ranganath Kini

**See Also:**: `JTextArea`

---

**Constructor Summary**

`TextPaneOutputStream(javax.swing.JTextPane textPane,
java.lang.String style)`
  
          Creates a new instance of TextAreaOutputStream which writes
to the specified instance of javax.swing.JTextArea control.


| **Method Summary** | |
| --- | --- |
| `void` | `write(int b)`             Writes the specified byte as a character to the javax.swing.JTextArea. |

**Methods inherited from class java.io.OutputStream**

`close, flush, write, write`

**Methods inherited from class java.lang.Object**

`clone, equals, finalize, getClass, hashCode, notify, notifyAll, toString, wait, wait, wait`

**Constructor Detail**

### TextPaneOutputStream

```
public TextPaneOutputStream(javax.swing.JTextPane textPane,
                            java.lang.String style)
```

:   Creates a new instance of TextAreaOutputStream which writes
    to the specified instance of javax.swing.JTextArea control.

    **Parameters:**: `textPane` - A reference to the javax.swing.JTextArea control to which the output must be redirected to.: `style` -


**Method Detail**

### write

```
public void write(int b)
           throws java.io.IOException
```

:   Writes the specified byte as a character to the
    javax.swing.JTextArea.

    :   **Specified by:**: `write` in class `java.io.OutputStream`
    :   **Parameters:**: `b` - The byte to be written as character to the JTextArea. **Throws:**: `java.io.IOException`


---


|  |  |  |
| --- | --- | --- |
| **Overview** \| **Package** \| **Class** \| **Use** \| **Tree** \| **Deprecated** \| **Index** \| **Help** | |  |
| **PREV CLASS**   NEXT CLASS | **FRAMES**    **NO FRAMES**     **All Classes** |  |
| SUMMARY: NESTED \| FIELD \| CONSTR \| METHOD | DETAIL: FIELD \| CONSTR \| METHOD |  |


---
